# *Curculigo recurvata* W.T.Aiton exhibits anti‐nociceptive and anti‐diarrheal effects in Albino mice and an in silico model

**DOI:** 10.1002/ame2.12119

**Published:** 2020-06-08

**Authors:** Shabbir Ahmad, Mst. Samima Nasrin, A. S. M. Ali Reza, Nishan Chakrabarty, Md. Akramul Hoque, Sanjida Islam, Mohammad Shah Hafez Kabir, Syed Mohammed Tareq, A. H. M. Khurshid Alam, Md. Areeful Haque, Md. Saiful Islam Arman

**Affiliations:** ^1^ Department of Pharmacy International Islamic University Chittagong Chittagong Bangladesh; ^2^ Department of Biochemistry & Molecular Biology University of Chittagong Chittagong Bangladesh; ^3^ Department of Chemistry Wayne State University Detroit MI USA; ^4^ Department of Pharmacy University of Rajshahi Rajshahi Bangladesh; ^5^ Drug and Herbal Research Centre, Faculty of Pharmacy Universiti Kebangsaan Malaysia Kuala Lumpur Malaysia

**Keywords:** analgesic, anti‐diarrheal, anti‐nociceptive, curculigine, *Curculigo recurvata*, isocurculigine

## Abstract

**Background:**

*Curculigo recurvata* (*C. recurvata*) is an enthnomedicinally important herb reported to have significant medicinal values. The present study aimed to explore the in vivo and in silico anti‐nociceptive and anti‐diarrheal effects of a *C. recurvate* rhizome methanol extract (Me‐RCR).

**Methods:**

The analgesic effects of Me‐RCR were assessed using acetic acid‐induced writhing and the formalin‐induced flicking test. The drugs were administered intraperitoneally (IP) at doses of 200 and 400 mg/kg body weight (bw). Anti‐diarrheal activity was evaluated by assessing intestinal motility, hypersecretion, and fecal score in mice at oral doses of 200 and 400 mg/kg·bw. Computer facilitated analyses for anti‐nociceptive and anti‐diarrheal activities of three isolated compounds from *C. recurvata* were undertaken to identify the best‐fit phytoconstituents.

**Results:**

The Me‐RCR showed significant (*P* < .05) peripheral anti‐nociception at the highest dose. The extract inhibited both early and late phases of nociception in the formalin‐induced writhing test. In the castor oil‐induced diarrhoea model, the extract significantly (*P* < .05) prolonged the onset time of diarrhoea, inhibited percentage of diarrhoea, and decreased both the volume and weight of intestinal contents. Rates of intestinal fluid accumulation inhibition were (33.61 ± 1.00)% and (46.44 ± 0.89)% at Me‐RCR doses of 200 and 400 mg/kg·bw, respectively. Moreover, a significant (*P* < .05) reduction in gastrointestinal motility was observed. An absorption, distribution, metabolism, excretion and/or toxicity (ADME/T) test showed that the selected compounds yielded promising results, satisfying Lipinski's rule of five for predicting drug‐like potential. Notably, of the three phytoconstituents curculigine and isocurculigine possessed the highest affinity for the COX‐1 and COX‐2. Isocurculigine was also identified as the most effective anti‐diarrheal compound in the computer‐facilitated model.

**Conclusion:**

An extract of the plant *C. recurvata* showed potential analgesic and anti‐diarrheal activity due to the presence of one or more active secondary metabolite(s).

## 1 INTRODUCTION

The use of herbal formulations as therapeutics in traditional medicine is widespread and historically ancient. Folk practitioners have long used such formulations based on tradition and experience with no proper scientific validation.^1^, ^2^ Therefore there is increasing need to bridge the gap between traditional uses of herbal preparations and scientifically generated natural products.

Pain is an unpleasant sensory and emotional response accompanying actual or potential tissue damage. The presence of pathologic conditions such as tumor, muscle spasm, inflammation, and nerve damage or exposure to noxious chemicals, or mechanical or thermal stimuli have been established as contributing factors of this damage.^3^ The injured tissue and migrating cells release various pro‐ and anti‐inflammatogenic agents including cytokines, interleukins, serotonin, histamine, prostaglandins, and nitric oxides that are responsible for regulation of the inflammation.^4^, ^5^ To relieve pain, opioid analgesics (morphine and codeine), non‐opioid analgesics (aspirin and diclofenac), and adjuvant analgesics are used to suppress the pain signal. However, their usage is limited by adverse effects on particular conditions such as peptic ulcers, heart failure, hypertension, and renal dysfunction, the allergic reactions caused by NSAIDs, and the state of tolerance and dependence induced by opioids.^1^, ^6^, ^7^

Diarrhea is associated with increased frequency of loose stools often accompanied by abdominal cramp. In many parts of the world, diarrhea has been shown to be a chronic disease or the result of infectious etiology.^8^ It is a common symptom of gastrointestinal infection due to ingestion of bacteria, viruses, or parasites transmitted by water, food, utensils, hands, and flies.^9^ The major causative agents of diarrhoea in humans include the *Shigella* species (*Shigella flexneri*, *Shigella sonnei*, *Shigella boydii*, *Shigella shiga*, and *Shigellla dysentery*), *Staphylococcus aureus*, *Escherichia coli* and *Salmonella typhi*.^10^ The World Health Organization (WHO) has encouraged the conduct of studies of the treatment and prevention of diarrhoeal diseases using traditional medical practices.^11^ Such efforts to find new medicinal agents with fewer toxic effects may lead to new drugs with improved pharmacological properties, thus substantially helping to extend the range of effective and safe therapies.

*Curculigo recurvata* (*C. recurvata*) W.T.Aiton (Family *Hypoxidaceae*) is a perennial herb found in the Chittagong Hill Tracts, and available in Cox's Bazar, Moulvibazar, and Sylhet in the marginal forest areas of Bangladesh. The genus, *Curculigo,* comprises 20 species. Some of these species are used by traditional practitioners and are believed to have significant medicinal values. The rhizomes of *C. recurvata* (RCR) are widely used in traditional medicine for several ailments such as snake bites, consumptive cough, impotence, asthma, jaundice, diarrhea, colic, gonorrhea, and arthropod stings,^12^, ^13^ as well as for the treatment of menoxenia, bleeding disorders, nephritis, arthritis, and leucorrhea.^13^, ^14^, ^15^ The traditional uses of RCR have been validated by animal and clinical studies, including hypoglycemic, antibacterial, anthelmintic,^16^ antithrombotic, and cytotoxic studies.^14^ Mustakim et al,^17^ for example, confirmed that a crude methanol extract of *C. recurvata* rhizomes possesses potential antioxidative and cytotoxic properties. Two novel diastereoisomer glucosides, curculigine and isocurculigine, have been identified from this species,^18^ and glucosides of curculigo rhizome have been shown to significantly improve premenopausal syndrome,^19^ characterized by hysteria, depression, and melancholia.^20^ Accumulating evidence suggests that other species of the *Curculigo* genus possess various therapeutic effects. For instance, the *C. orchioides* has shown neuroprotective,^21^, ^22^ anticancer,^22^ antiosteoporotic,^24^ immunostimulatory,^25^ and estrogenic activities.^26^

Traditional usage in the treatment of several diseases and accumulating pharmacologic evidence suggest that *C. recurvata* is a plant with rich potential for therapeutic studies. The folkloric value of *C. recurvata* encouraged us to investigate the pharmacological activity of this plant in this study. The objective of the study was to assess the anti‐nociceptive and anti‐diarrheal activities of a methanol extract of *C. recurvata* (Me‐RCR), in order to determine its relevance for the treatment of pain and diarrhea.

## 2 MATERIALS AND METHODS

### 2.1 Chemicals and reagents

Formalin and acetic acid were obtained from Merck (India). DMSO and methanol were from Merck (Germany). Loperamide was from Square Pharmaceuticals Ltd, Bangladesh. Castor oil was from WELL’s Heath Care, Spain. Diclofenac‐Na was from Sigma‐Aldrich, USA. Analytical reagent grade chemicals were used for all experiments.

### 2.2 Plant materials

RCR was collected from Chittagong Hill Tracts area, Bangladesh. The plants were identified by Dr Shaikh Bokhtear Uddin, Taxonomist and Professor, Department of Botany, University of Chittagong. A voucher specimen (Accession No. 16012) has been deposited at the Department of Pharmacy, International Islamic University Chittagong, Chittagong, Bangladesh.

### 2.3 Preparation of crude extract

The RCR was air dried (23 ± 0.5)°C followed by mechanical drying (Ecocell, MMM Group, Germany) at 55‐60°C. The dried rhizomes were ground into a coarse powder with a mechanical grinder (NOWAKE, Japan). The powdered rhizome (500 gm) was soaked in methanol (800 mL) for 7 days with occasional shaking and then filtered using Whatman #1 filter paper (Whatman International, Maidstone, Kent, UK). The filtrate was then evaporated under reduced pressure at 50°C using a rotary evaporator (RE200, Bibby Sterling Ltd, UK) to yield a brown mass of 35.5 gm of crude methanolic extract of RCR (Me‐RCR). The extract was stored in a freezer until used for the experiments.

### 2.4 Experimental animals

Swiss albino mice were purchased from International Center for Diarrheal Diseases Research, Dhaka, Bangladesh (ICDDRB). The animals were acclimatized for 7 days in the laboratory environment prior to the study. The study was conducted following approval by the Institutional Animal Ethical Committee, Department of Pharmacy, International Islamic University Chittagong, Bangladesh, according to governmental guidelines.^27^ The protocol in this study used mice as the animal model for diarrhea and pain research and was approved by the Institutional Animal Ethical Committee. This research work was approved by the Ethical Review Committee of the Department of Pharmacy, International Islamic University Chittagong, Bangladesh (ref. Pharm/P&D/2017/01).

### 2.5 Phytochemical screening

A qualitative phytochemical analysis of Me‐RCR was performed to check for the presence of secondary metabolites, particularly saponins, flavonoids, steroids, alkaloids, carbohydrates, terpenoids, tannins, quinine, cellulose, and glycosides.^6^

### 2.6 Anti‐nociceptive activity

#### 2.6.1 Acetic acid‐induced writhing test

Twenty four mice were divided into 4 groups (n = 6). The first group, the normal control (NC), received normal saline (10 mL/kg·bw), the second group, the reference control (DS), received the standard drug diclofenac sodium (10 mg/kg·bw), and the remaining two groups received Me‐RCR at doses of 200 and 400 mg/kg·bw. Thirty minutes after saline, diclofenac sodium or plant extract injection, the animals were treated IP with 10 mL/kg·bw of acetic acid (1% (v/v)). After 5 min of acetic acid injection, abdominal constrictions were counted for 10 min and the responses were compared with control group.^28^, ^29^ Inhibition of writhing was calculated using the following formula:

$$\text{Writhing inhibition}=\frac{\text{Mean no}.\;\text{of writhing}\left(\text{control}\right)-\text{Mean no}.\;\text{of writhing}\left(\text{test}\right)}{\text{Mean number of writhing}\left(\text{control}\right)}\times 100.$$

#### 2.6.2 Formalin‐induced licking test

The formalin‐induced biphasic method used in mice models has been described previously.^29^, ^30^ A volume of formalin solution (2.5%, 20 µL) was prepared by 0.9% saline solution and injected into the sub‐plantar region of the right hind paw of mice to induce pain. Animals were pretreated by IP injection of vehicle (saline water), diclofenac sodium (10 mg/kg·bw), and different doses (200 and 400 mg/kg·bw) of Me‐RCR 60 minutes before formalin injection. Responses such as licking and biting of the right hind paw were considered as nociception. Responses measured during the first 5 minutes after formalin injection were considered as the first phase and responses 15‐30 minutes after injection were considered as the second phase. First and second phase responses were judged to describe neurogenic and inflammatory pain, respectively. Anti‐nociceptive activity was calculated as the percentage inhibition of licking time.

$$\text{Percentage inhibition}=\frac{\text{Mean of licking time}\left(\text{control}\right)-\text{Mean of licking time}\left(\text{test}\right)}{\text{Mean of licking time}\left(\text{control}\right)}\times 100.$$

### 2.7 Anti‐diarrheal activity

#### 2.7.1 Castor oil‐induced diarrhea

The anti‐diarrheal effects of the Me‐RCR were determined according to the method described by Taufiq and Bellah.^6^, ^31^ Swiss albino mice were fasted for 24 hours and divided into 4 groups (n = 6). The first group received 10 mL/kg·bw of Tween 80 (1% Tween 80 in water) orally as the normal control (NC), the second group received loperamide (5 mg/kg·bw as oral suspension) as the reference control (RC) and the remaining two groups received Me‐RCR orally at doses of 200 and 400 mg/kg·bw. Oral administration of castor oil (0.5 mL) was done after 1 h of administration of samples. The mice were placed in separate cages lined with transparent paper, which was changed every hour for better visibility of fecal materials. Counting of droppings was done at 60 min intervals for 4 hours. The average number of stools passed by treated groups was compared with that of the NC and RC groups. The mean number of diarrheic faeces pooled by the control group was considered as 100%. The level of inhibition (%) of defecation caused by Me‐RCR treatment was calculated relative to the control using the following equation:

$$\text{Inhibition of defecation}\left(\%\right)=\left[\left(\text{NDC}-\text{NDT}\right)/\text{NDC}\right]\times 100,$$

where NDC = mean number of diarrheic faeces of the control group and NDT = mean number of diarrheic faeces of the treated group.

#### 2.7.2 Castor oil‐induced enteropooling

The castor oil‐induced enteropooling test was performed using the method described previously by Robert.^32^ Swiss albino mice were fasted for 24 hours with free access to water and divided into 4 groups (n = 6). The first group received 10 mL/kg·bw of Tween 80 (1% Tween 80 in water) orally as the normal control (NC), the second group received loperamide (5 mg/kg·bw as an oral suspension) as the reference control (RC) and the remaining two groups received Me‐RCR orally at doses of 200 and 400 mg/kg·bw. Castor oil was given orally 1 hour after administration of the samples. The mice were sacrificed 2 hours later using 70% (v/v) ethanol in 0.9% sterile saline as an anesthetic. After ligation at the pyloric sphincter and ileocecal junctions, the intervening portion of small intestine was dissected out and weighed. The intestines were emptied and contents were collected into a graduated tube and the volume of the content was measured. The intestines were reweighed and the difference between full and empty intestines was calculated.

#### 2.7.3 Gastrointestinal motility test

This test was carried out by the method described by Mascolo.^33^ Swiss albino mice were fasted for 24 hours with free access to water and divided into 4 groups (n = 6). The first group received 10 mL/kg·bw of Tween 80 (1% Tween 80 in water) orally as the normal control (NC), the second group received loperamide (5 mg/kg·bw as oral suspension) as the reference control (RC) and the remaining two groups received Me‐RCR orally at doses of 200 and 400 mg/kg·bw. One hour after treatment, the animals received 1 mL of charcoal meal (10% charcoal suspension in 5% gum acacia) orally. After 1 hour, the animals were sacrificed using 70% (v/v) ethanol in 0.9% sterile saline as an anesthetic and the distance traveled by the charcoal meal from pylorus to caecum was measured and expressed as a percentage of the total distance of the intestine. Percentage inhibition was calculated using the following formula:

$$\text{Percentage inhibition}=\frac{\text{Distance travelled by the control}-\text{Distance travelled by the test}}{\text{Distance travelled by the control}}\times 100.$$

### 2.8 Selection of compounds for PASS prediction

The three compounds curculigine, isocurculigine, and nyasicoside were selected for PASS prediction analysis on the basis of availability, as the main compounds isolated from RCR identified by a literature review.^18^ The chemical structures of the selected compounds were obtained from the PubChem data base.

### 2.9 In silico experiment to predict the activity spectra for substances (PASS)

The suitability of the selected compounds (curculigine, isocurculigine, and nyasicoside)^18^ for studying anti‐nociceptive activity was confirmed using the PASS prediction program. This program predicts the theoretical biological or pharmacological activity of a compound as probable activity (P_a_) and probable inactivity (P_i_)^29^ based on the results of analysis of the structure‐activity relationship of 205 000 candidate compounds, predicting more than 3750 kinds of biological activities. The range of values of Pa and Pi lies between 0.000 and 1.000. When Pa > Pi, a compound is considered to be experimentally active. High pharmacological potential is probable at Pa > 0.7 and a Pa between 0.5 and 0.7 indicates noticeable experimental pharmacological potential. Pa < 0.5 implies low pharmacological activity but with some chance of finding a new biologically active compound.^34^, ^35^

### 2.10 In silico molecular docking

#### 2.10.1 Preparation of protein

The structures of cyclooxygenase‐1 (COX‐1, PDB id: 2OYE), cyclooxygenase‐2 (COX‐2, PDB id: 3HS5) and M3 muscarinic acetylcholine receptor (PDB id: 4U14 and PDB id: 5AIN) were obtained in PDB format from the protein data bank.^36^ The structure was then prepared and refined using the Protein Preparation Wizard program (Schrödinger‐Maestro v10.1). Charges and bond orders were assigned while hydrogens were added to the heavy atoms and selenomethionines were converted to methionines followed by deleting all water molecules. Minimization was done by setting the value of RMSD (root‐mean‐square‐deviation) to 0.30 Å for maximum heavy atoms using force field OPLS_2005.

#### 2.10.2 Ligand preparation

The structures of the selected compounds (curculigine, isocurculigine, and nyasicoside) were obtained from Pubchem databases. Ligprep 2.5 within the Schrödinger Suite 2015 utilizing OPLS_2005 force field was used to build the 3D model of the ligands. Various ionization states of the compounds were generated at pH (7.0 ± 2.0) using Epik 2.2 from the Schrödinger Suite. Up to 32 possible stereoisomers were retained per ligand for analysis.

#### 2.10.3 Receptor grid generation

Receptor grids were measured for prepared proteins so that different ligand poses could bind within the predicted active site during docking. In glide, grids were generated in so as to keep the default parameters of Van der Waals scaling factor and the charge cutoff value as 1.00 0.25, respectively, subject to OPLS 2005 force field. A cubic box of specific dimensions centered on the centroid of the active site residues was generated as a receptor. The bounding box was set to 14 Å × 14 Å × 14 Å for docking experiments.

#### 2.10.4 Glide Standard Precision (SP) ligand docking

Standard precision flexible ligand docking was performed using Schrödinger‐Maestro v 10.1,^37^, ^38^ within which penalties were applied to non‐cis/trans amide bonds. Values were set as 0.15 and 0.80 for Van der Waals partial charge cutoff and scaling factor, respectively, for ligand atoms. Finally, glide score was calculated through energy‐minimized poses and for each ligand the significant docked pose with the lowest glide score was documented.

### 2.11 Absorption, Distribution, metabolism, excretion and/or toxicity (ADME/T) property analysis

The pharmacokinetic parameters or drug‐likeness properties of the isolated compounds were determined by QikProp, a module of Schrodinger (Maestro, version 10.1). This program can predict pharmacokinetic and physicochemical properties including molecular weight of the compounds, Lipophilicity (LogP), the number of hydrogen‐bond acceptors, the number of hydrogen‐bond donors and molar refractivity. Lipinski's rule of five was used to determine drug‐like activity of ligand molecules. ADME/T properties of the compound (DIM) were analyzed using Qikprop 3.2 module.^39^

### 2.12 Statistical analysis

Data are expressed as means ± SEM. One‐way ANOVA followed by a post‐hoc test (Dunnet's *t* test) was used to find significant differences between test and control groups. GraphPad Prism Version 6.0 (GraphPad software Inc, San Diego, CA) was used for the statistical analysis. *P* values (<.05 and <.001) were considered as statistically significant.

## 3 RESULTS

### 3.1 Phytochemical screening

Qualitative phytochemical tests revealed the presence of carbohydrates, flavonoids, tanins, saponins, and cardiac glycosides (Table 1).

**Table 1:** Qualitative phytochemical screening of Me‐RCR extract

Phytochemical class	Test performed	Observations	Results
Alkaloids	Dragendorff's test	Turbidity/precipitation	−
Carbohydrates	Molisch test	Formation of a purple product at the interface of the two layers	+
Flavonoid	Ferric chloride test	Formation of yellow color which changed to colorless on acid addition	+++
Steroids	Liebermann Burchard test	Green to pink color was absent	−
Terpenoids	Liebermann Burchard test	Appearance of reddish brown‐deep red color	−
Cardiac Glycosides	Keller Killanic test	Lower reddish brown layer & upper acetic acid layer which turns bluish green	+
Tannins	Gelatin test	Appearance of white precipitate	++
Saponins	Froth test	Stable froth formation	+

Note

Bioavailability Key.

−, not present; +, present in low concentration; ++, present in moderately high concentration; +++, present in very high concentration.

### 3.2 Anti‐nociceptive activity

#### 3.2.1 Acetic acid‐induced writhing test

**Figure 1:**
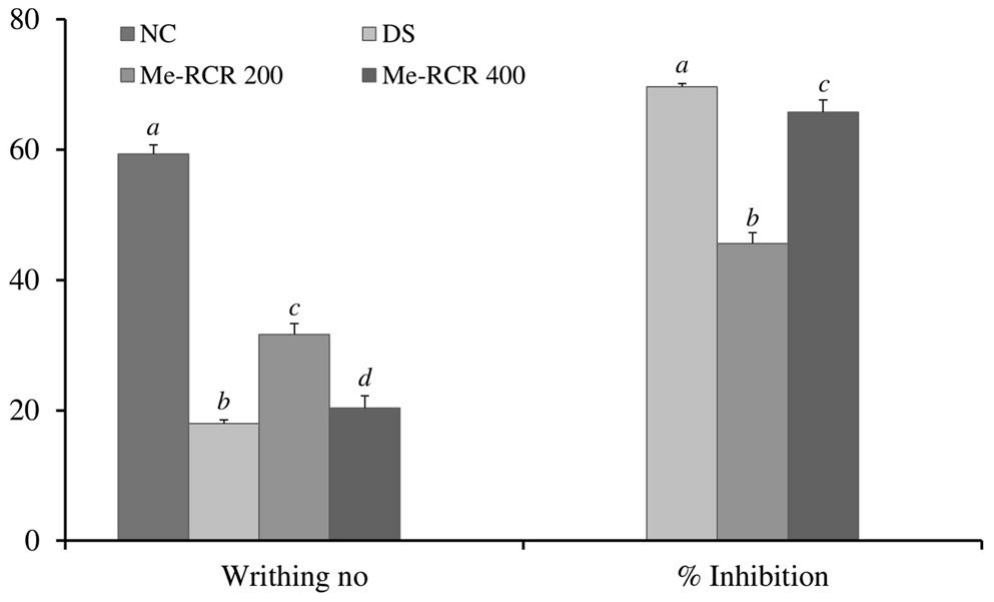
Effect of the Me‐RCR extract on acetic acid‐induced writhing response in mice. Data are shown as means ± SEM (n = 6). ^abc^Values with different superscript letters for a given time period are significantly different from other groups of animals (Dunnet's test for multiple comparisons, *P* < .05). NC, normal control; DS, diclofenac sodium; Me‐RCR 200, methanol extract of *C. recurvata* at 200 mg/kg; Me‐RCR 400, methanol extract of *C. recurvata* at 400 mg/kg

Me‐RCR showed significant (*P* < .05) anti‐nociceptive activity in the acetic acid‐induced writhing test (Figure 1). Me‐RCR at doses of 200 and 400 mg/kg·bw and the reference standard diclofenac sodium (DS) at a dose of 10 mg/kg·bw showed inhibitory effects of (45.63 ± 1.43)%, (65.73 ± 1.05)% and (69.66 ± 1.38)%, respectively.

#### 3.2.2 Formalin‐induced licking test

**Figure 2:**
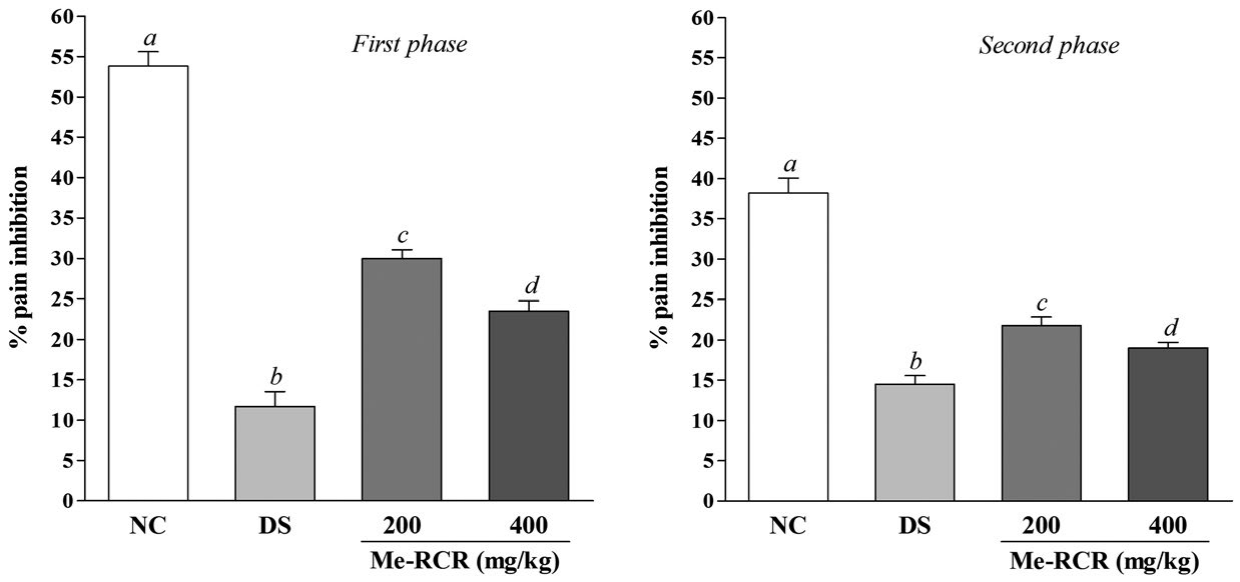
Effects of the Me‐RCR extract on hind paw licking in the formalin test in mice. Data are shown as means ± SEM (n = 6). ^abc^Values with different superscript letters over the lines for a given time period are significantly different from other groups of animals (Dunnet's test for multiple comparisons, *P* < .05). DS, diclofenac sodium; Me‐RCR 200, methanol extract of *C. recurvata* at 200 mg/kg; Me‐RCR 400, methanol extract of *C. recurvata* at 400 mg/kg; NC, normal control

The biphasic nociceptive potential of Me‐RCR against the formalin‐induced pain test is shown in Figure 2. Me‐RCR showed significant (*P* < .05) anti‐nociceptive action induced by formalin. In the early phase, the Me‐RCR attenuated nociception to (29.95 ± 1.10)% and (23.52 ± 1.26)% at the doses of 200 and 400 mg/kg·bw, respectively, whereas the DS standard showed (11.69 ± 1.84)% pain inhibition. In the late phase, Me‐RCR reduced the licking time by (21.78 ± 1.06)% and (19.02 ± 0.69)% of inhibition at doses of 200 and 400 mg/kg·bw, respectively; in contrast, the DS elicited (14.53 ± 1.03)% nociceptive inhibition.

### 3.3 Anti‐diarrheal activity

#### 3.3.1 Castor oil‐induced diarrhea test

**Figure 3:**
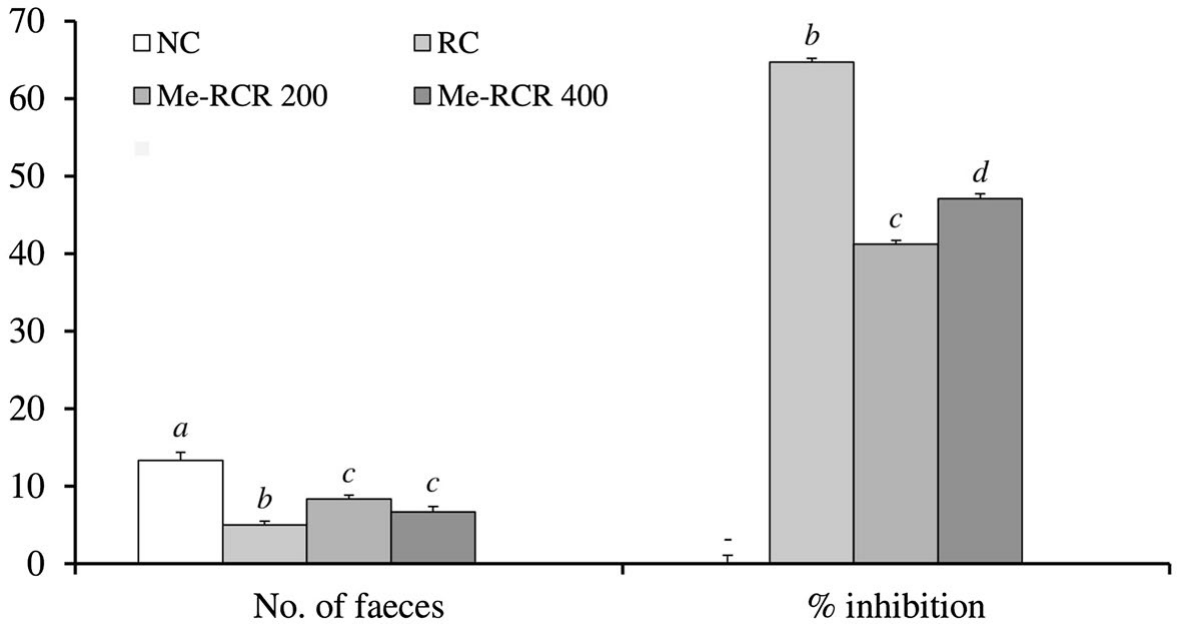
Effect of the Me‐RCR extract on castor oil‐induced diarrhea in mice. All values are expressed as means ± SEM (n = 6); Data were analyzed by one‐way analysis of variance (ANOVA) followed by Dunnet's test for multiple comparisons. Values with *P* < .05 were considered as significant. ^a‐d^Different superscript letters above the bars are significantly different from each other. Me‐RCR 200, methanol extract of *C. recurvata* at 200 mg/kg; Me‐RCR 400, methanol extract of *C. recurvata* at 400 mg/kg; NC, normal control; RC, reference control (loperamide)

Me‐RCR significantly (*P* < .05) reduced the frequency of defecation and the number of faeces at doses of 200 and 400 mg/kg·bw compared to the control. Percentage diarrheal inhibition values were (41.18 ± 1.21)% and (47.06 ± 1.09)% at doses of 200 mg/kg and 400 mg/kg, respectively (Figure 3). The standard loperamide achieved a percentage diarrheal inhibition of (67.71 ± 1.02)% at a dose of 5 mg/kg·bw.

#### 3.3.2 Castor oil‐induced enteropooling in mice

Castor oil (also known as recinolic acid) has a tendency to produce diarrhea through accumulation of fluid in the intestinal loop. In the gastrointestinal enteropooling test, Me‐RCR significantly (*P* < .05) decreased the volume as well as the weight of intestinal content dose dependently. Percentage inhibition of intestinal fluid accumulation was (33.61 ± 1.00)% and (46.44 ± 0.89)% at doses of 200 and 400 mg/kg·bw, respectively (Table 2). In addition, the weight of intestinal content was reduced by (0.41 ± 0.01) g and (0.35 ± 0.01) g at the same doses. Loperamide inhibited the percentage of intestinal fluid accumulation by (51.37 ± 2.15)% and reduced the weight of intestinal content to (0.30 ± 0.01) gm.

**Table 2:** Effect of Me‐RCR extract on castor oil‐induced enteropooling in mice

Group	Treatment	Volume of intestinal content (ml)	Weight of intestinal content (g)	% Inhibition of intestinal content
I	NC	0.72 ± 0.04	0.61 ± 0.03	—
II	RC	0.34 ± 0.02**	0.30 ± 0.01***	51.37 ± 2.15
III	Me‐RCR 200	0.47 ± 0.01*	0.41 ± 0.01**	33.61 ± 1.00
IV	Me‐RCR 400	0.40 ± 0.01**	0.35 ± 0.01***	46.44 ± 0.89

Note

Values are mean ± SEM (n = 6).

Dunnett test as compared to normal control (NC). Statistical representation of the weight of intestinal content (g) by Me‐RCR, positive anti‐diarrheal control (Loperamide, 5 mg/kg PO) processed by paired t‐test analysis (Dennett's test). Data were analyzed by one‐way Analysis of Variance (ANOVA) using GraphPad Prism for Windows, Version 6.0.

Abbreviations: Me‐RCR 200, methanol extract of *C. recurvata* at 200 mg/kg; Me‐RCR 400, methanol extract of *C. recurvata* at 400 mg/kg; NC, normal control; RC, loperamide.

* *P* < .05.

** *P* < .01.

*** *P* < .001.

#### 3.3.3 Gastrointestinal motility test

Me‐RCR significantly (*P* < .001) reduced the gastrointestinal transit of marker diet in castor oil‐induced mice at all doses (Table 3). Maximum effect (43.01%) was achieved at a dose of 400 mg/kg·bw. Loperamide (5 mg/kg·bw) produced a similar decrease in gastrointestinal movement (42.84%).

**Table 3:** Effect of Me‐RCR extract on charcoal induced gut transit changes in Swiss albino mice

Group	Treatment	% of intestine cross by marker	% of inhibition
I	NC	83.18 ± 2.91	—
II	RC	45.95 ± 1.40***	42.84
III	Me‐RCR 200	60.55 ± 0.63*	24.69
IV	Me‐RCR 400	45.82 ± 0.94***	43.01

Note

Values are mean ± SEM (n = 6).

Dunnett test as compared to normal control (NC). Statistical representation of the % of intestine cross by marker by Me‐RCR extract, positive anti‐diarrheal control (loperamide) processed by paired t‐test analysis (Dennett's test). Data were analyzed by one‐way Analysis of Variance (ANOVA) using GraphPad Prism for Windows, Version 6.0.

Abbreviations: Me‐RCR 200, methanol extract of *C. recurvata* at 200 mg/kg; Me‐RCR 400, methanol extract of *C. recurvata* at 400 mg/kg; NC, normal control; RC, loperamide.

* *P* < .05;

*** *P* < .001.

### 3.4 In silico PASS prediction

The compounds curculigine, isocurculigine, and nyasicoside were evaluated by the PASS program for their anti‐nociceptive effects. All the compounds demonstrated a higher P_a_ than P_i_ (Table 4). Curculigine and isocurculigine showed the highest P_a_ value for anti‐nociceptive activity (P_a_ = 0.480), followed by nyasicoside (P_a_ = 0.454).

**Table 4:** PASS prediction of anti‐nociceptive activity of curculigine, isocurculigine, and nyasicoside

Phytocompounds with their chemical structures		PASS prediction of anti‐nociceptive activity	
		Pa	Pi
Curculigine	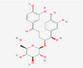	0.480	0.049
Isocurculigine	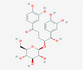	0.480	0.049
Nyasicoside	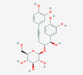	0.454	0.067

### 3.5 In silico molecular docking analysis for analgesic activity

**Figure 4:**
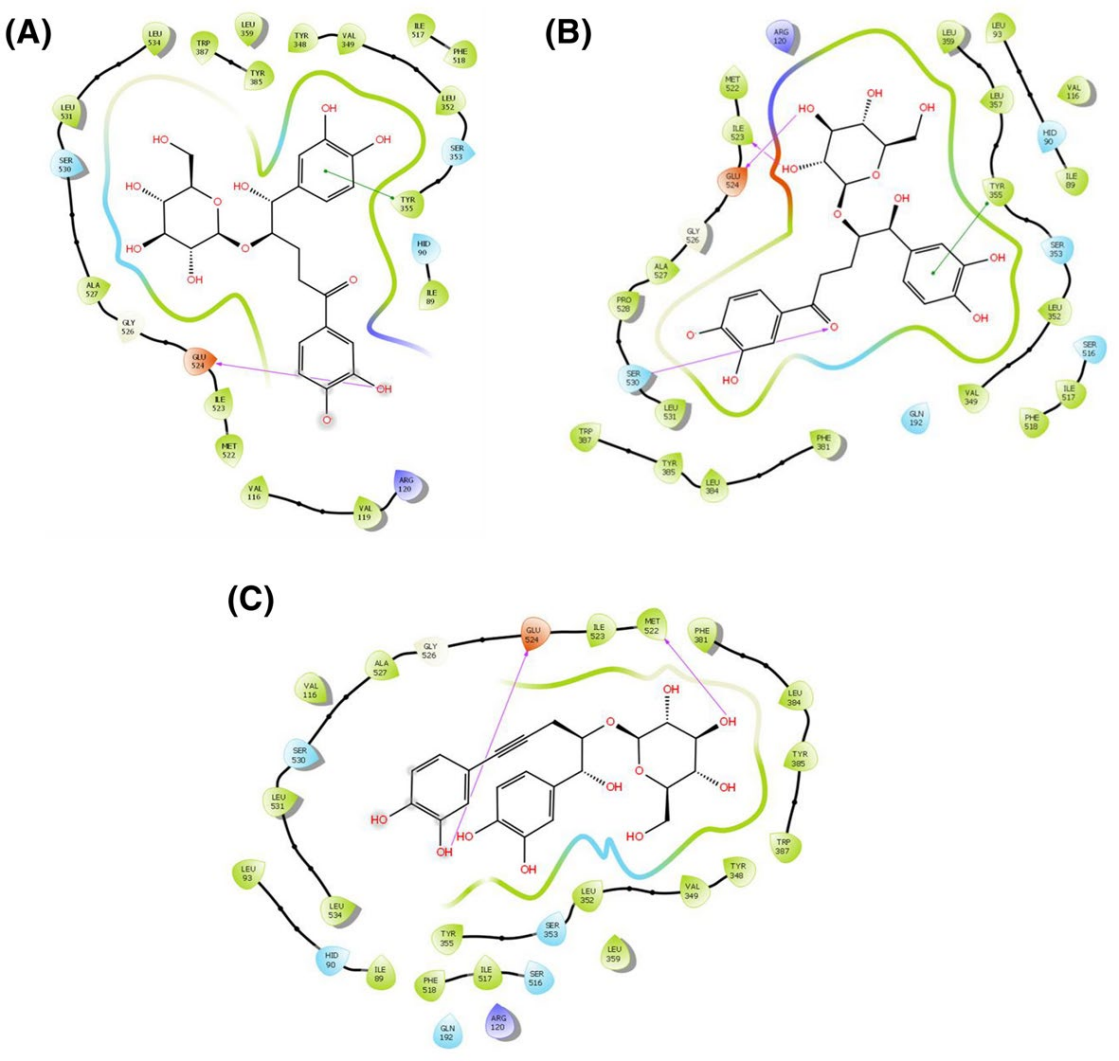
Docking analysis for analgesic effect of curculigine (A), isocurculigine (B) and nyasicoside (C) docking with COX 1 (PDB: 2OYE). The colors indicate the residue (or species) type: red: acidic (Asp, Glu); green: hydrophobic (Ala, Val, Ile, Leu, Tyr, Phe, Trp, Met, Cys, Pro); purple: basic (Hip, Lys, Arg); blue: polar (Ser, Thr, Gln, Asn, His, Hie, Hid); light gray: other (Gly, water); darker gray: metal atoms. Interactions with the protein are marked with lines between ligand atoms and protein residues: solid pink: H‐bonds to the protein backbone; dotted pink: H‐bonds to protein side chains; green: π‐π stacking interactions; orange: π‐cation interactions. Ligand atoms that are exposed to solvent are marked with gray spheres. The protein “pocket” is displayed with a line around the ligand, colored with the color of the nearest protein residue. The gap in the line shows the opening of the pocket

**Figure 5:**
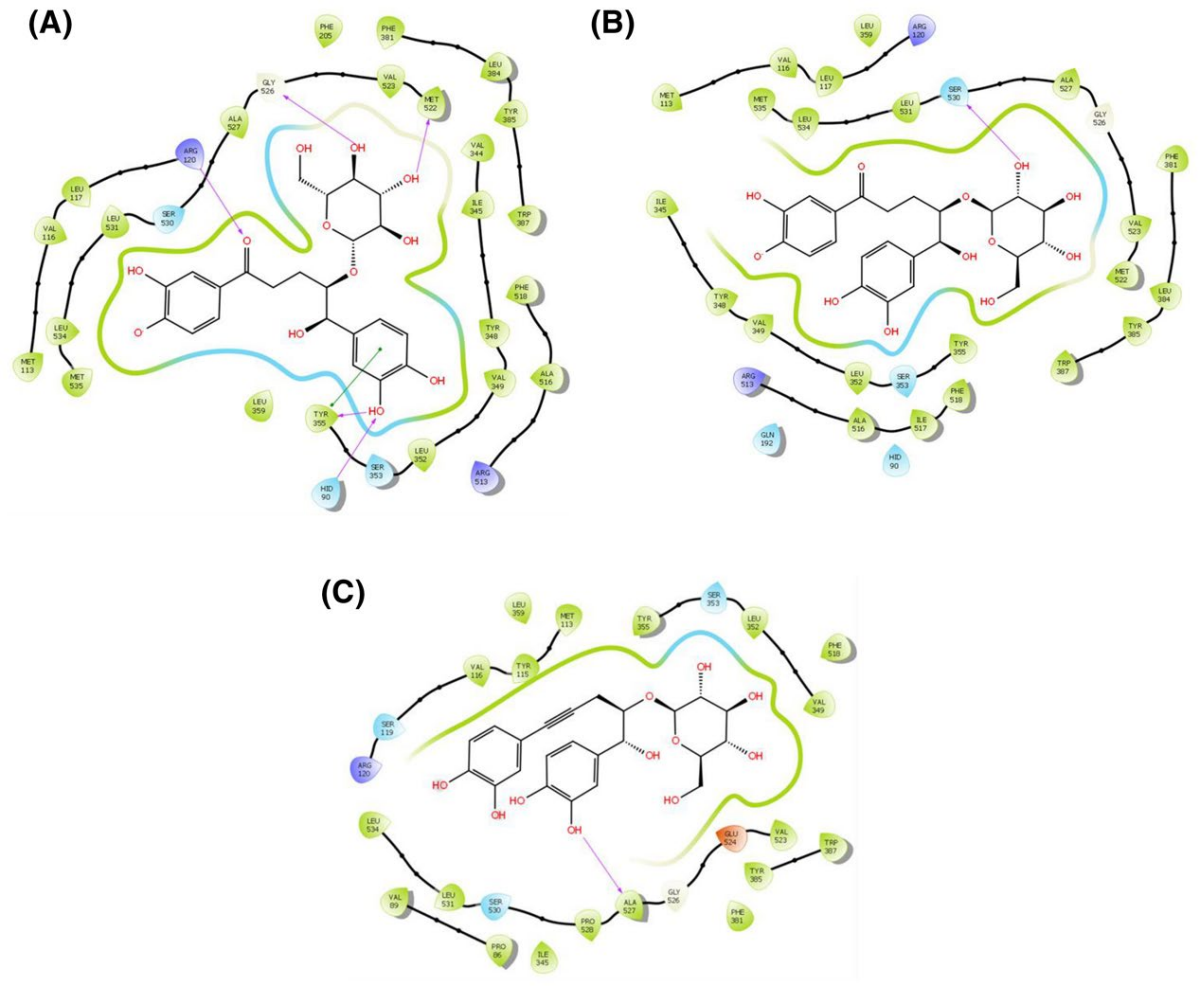
Docking analysis for analgesic effect of curculigine (A), isocurculigine (B) and nyasicoside (C) docking with COX 2 (PDB: 3HS5). The colors indicate the residue (or species) type: red: acidic (Asp, Glu); green: hydrophobic (Ala, Val, Ile, Leu, Tyr, Phe, Trp, Met, Cys, Pro); purple: basic (Hip, Lys, Arg); blue: polar (Ser, Thr, Gln, Asn, His, Hie, Hid); light gray: other (Gly, water); darker gray: metal atoms. Interactions with the protein are marked with lines between ligand atoms and protein residues: solid pink: H‐bonds to the protein backbone; dotted pink: H‐bonds to protein side chains; green: π‐π stacking interactions; orange: π‐cation interactions. Ligand atoms that are exposed to solvent are marked with gray spheres. The protein “pocket” is displayed with a line around the ligand, colored with the color of the nearest protein residue. The gap in the line shows the opening of the pock

A computational study was performed to identify the virtual analgesic potential of the molecules. A grid and ligand based molecular docking program was used to assess the binding pattern of molecules with the amino acids present in the active pocket of the protein. The study showed the docking of the identified active chemical compounds of *C. recurvata* to the active sites of the cyclooxygenase enzymes (COX‐1 and COX‐2). The interactions of the selected compounds with 2OYE and 3HS5 were studied using the Schrodinger suite v10.1. Of the three compounds, curculigine and isocurculigine showed the highest docking scores (Tables 5 and 6). A low and negative free energy value for binding indicates a strong favorable bond between 2OYE and 3HS5. Curculigine and isocurculigine had docking scores of − 10.434 and − 8.868 against the COX‐1 and COX‐2 enzymes, respectively. The results of the docking analysis were depicted in Figures 4 and 5.

**Table 5:** Docking analysis to assess analgesic effect of curculigine, isocurculigine, and nyasicoside docking with COX 2 (PDB: 3HS5)

Compound's name	Docking score	Glide e model	Glide energy
Curculigine	−6.931	−24.527	−24.75
Isocurculigine	−8.868	−44.008	−28.914
Nyasicoside	−6.876	−41.244	−43.608

**Table 6:** Docking analysis to assess analgesic effect of curculigine, isocurculigine, and nyasicoside docking with COX 1 (PDB: 2OYE)

Compound's name	Docking score	Glide e model	Glide energy
Curculigine	−10.434	−86.917	−68.696
Isocurculigine	−8.477	−62.259	−50.923
Nyasicoside	−9.461	−75.961	−57.893

### 3.6 In silico molecular docking analysis for anti‐diarrheal activity

**Figure 6:**
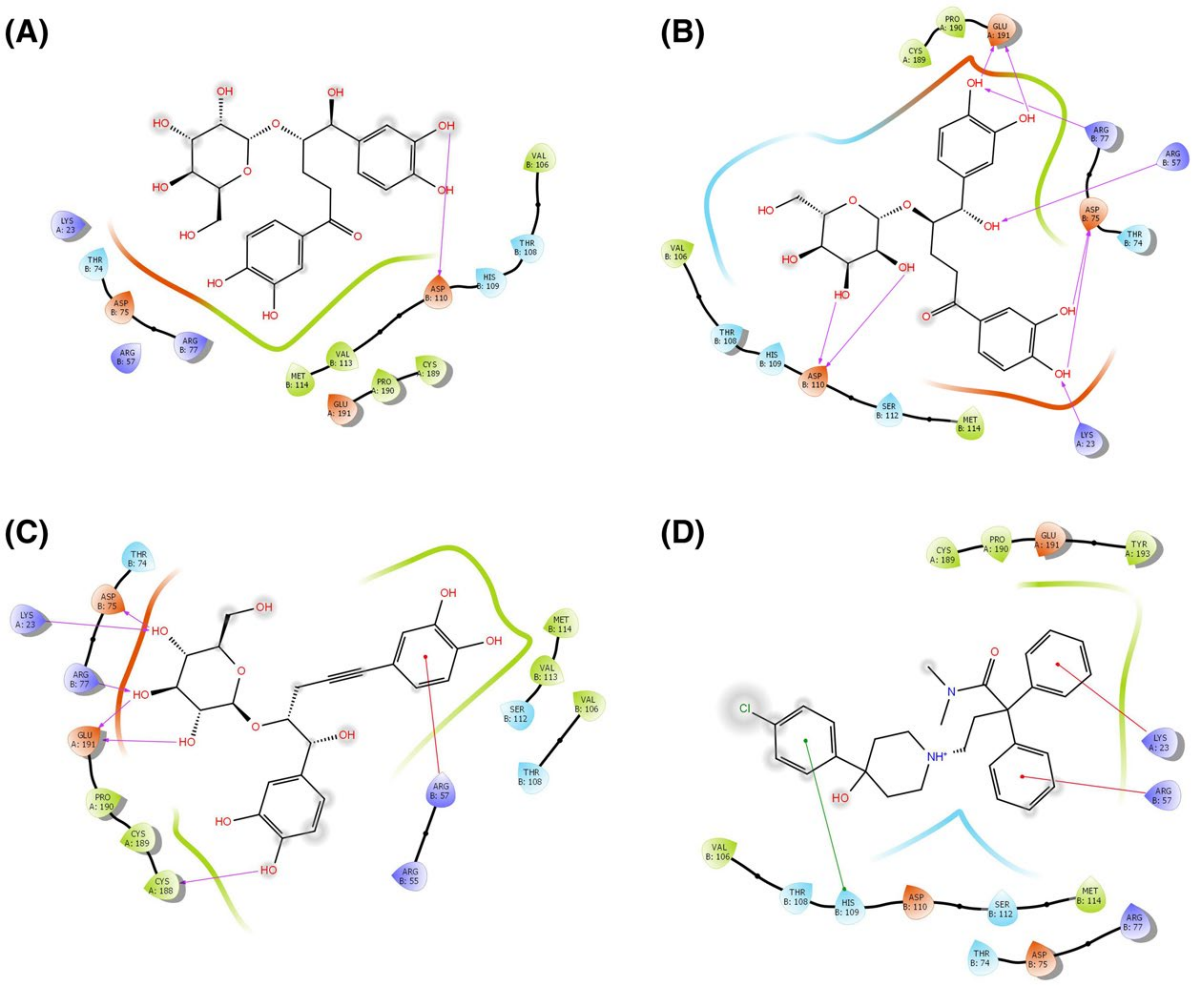
Docking analysis for anti‐diarrheal activity of curculigine (A), isocurculigine (B), nyasicoside (C) and loperamide (D) docking with M3 muscarinic receptor (PDB: 5AIN). The colors indicate the residue (or species) type: red: acidic (Asp, Glu); green: hydrophobic (Ala, Val, Ile, Leu, Tyr, Phe, Trp, Met, Cys, Pro); purple: basic (Hip, Lys, Arg); blue: polar (Ser, Thr, Gln, Asn, His, Hie, Hid); light gray: other (Gly, water); darker gray: metal atoms. Interactions with the protein are marked with lines between ligand atoms and protein residues: solid pink: H‐bonds to the protein backbone; dotted pink: H‐bonds to protein side chains; green: π‐π stacking interactions; orange: π‐cation interactions. Ligand atoms that are exposed to solvent are marked with gray spheres. The protein “pocket” is displayed with a line around the ligand, colored with the color of the nearest protein residue. The gap in the line shows the opening of the pocket

**Figure 7:**
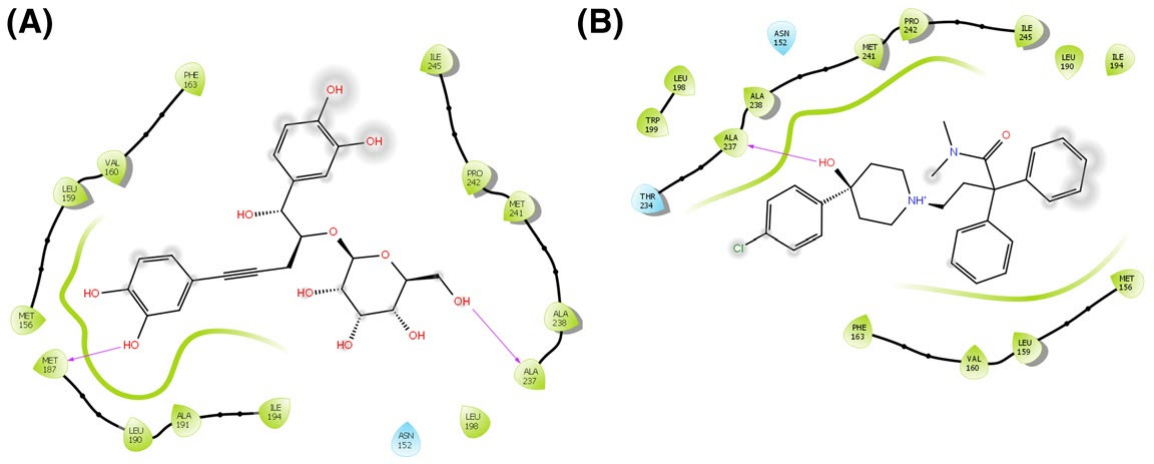
Docking analysis for anti‐diarrheal activity of nyasicoside (C) and loperamide (D) docking with M3 muscarinic receptor (PDB: 4U14). The colors indicate the residue (or species) type: red: acidic (Asp, Glu); green: hydrophobic (Ala, Val, Ile, Leu, Tyr, Phe, Trp, Met, Cys, Pro); purple: basic (Hip, Lys, Arg); blue: polar (Ser, Thr, Gln, Asn, His, Hie, Hid); light gray: other (Gly, water); darker gray: metal atoms. Interactions with the protein are marked with lines between ligand atoms and protein residues: solid pink: H‐bonds to the protein backbone; dotted pink: H‐bonds to protein side chains; green: π‐π stacking interactions; orange: π‐cation interactions. Ligand atoms that are exposed to solvent are marked with gray spheres. The protein “pocket” is displayed with a line around the ligand, colored with the color of the nearest protein residue. The gap in the line shows the opening of the pocket

Computer aided anti‐diarrheal activity was performed to assess the binding pattern of molecules with the amino acids present in the active pocket of the protein. In this study, two major receptors (M3 muscarinic acetylcholine receptor, PDB: 4U14 and PDB: 5AIN) involved in intestinal motility were used to explore the possible anti‐diarrheal activity of Me‐RCR. Of the three compounds, isocurculigine showed the highest docking score (−4.22) against the 5AIN receptor, which was higher than that of the standard loperamide (−2.41), whereas, the nyasicoside showed the highest docking score (−4.31) against 4U14 receptors (Table 7). The results of the docking analysis are depicted in Figures 6 and 7.

**Table 7:** Docking analysis to assess anti‐diarrheal activity of curculigine, isocurculigine, nyasicoside, and loperamide docking with M3 muscarinic acetylcholine receptor (PDB: 5AIN and 4U14)

Compound's name	Docking score		Glide emodel		Glide energy	
	5AIN	4U14	5AIN	4U14	5AIN	4U14
Curculigine	−1.646	—	20.307	—	26.973	—
Isocurculigine	−4.224	—	58.947	—	47.894	—
Nyasicoside	−3.682	−4.311	43.138	−44.315	38.272	−36.411
Loperamide	−2.414	−5.324	37.291	−45.061	33.577	−36.406

### 3.7 ADME/T property analysis

The absorption, distribution, metabolism and excretion (ADME) properties of curculigine, isocurculigine, and nyasicoside were clarified with the QikProp module of Schrodinger (Table 8). The selected properties are known to be cell permeability, metabolism, and bioavailability. The predicted properties of curculigine, isocurculigine, and nyasicoside showed promising results, satisfying Lipinski's rule of five for identification of drug like potential.

**Table 8:** ADME/T properties of curculigine, isocurculigine, and nyasicoside by QikProp

Name of molecules	PubChem ID	MW^a^	HB donor^b^	HB acceptor^c^	Log P^d^	Molar refractivity^e^
Curculigine	101664510	496.46	9	16.9	−2.117	118.34
Isocurculigine	101664511	496.46	9	16.9	−2.121	118.34
Nyasicoside	10648327	478.45	9	14.9	−1.283	116.08

^a^ Molecular weight (acceptable range: <500).

^b^ Hydrogen bond donor (acceptable range: ≤5).

^c^ Hydrogen bond acceptor (acceptable range: ≤10).

^d^ High lipophilicity (expressed as LogP, acceptable range: ˂5).

^e^ Molar refractivity should be between 40‐130.

## 4 DISCUSSION

Natural products obtained from plants have an important role in the management of a wide range of diseases. New effective and safe analgesic and anti‐inflammatory drugs may be found by screening medicinal plants that have traditional analgesic and anti‐inflammatory uses,^40^, ^41^ and efforts should be made to enrich drug arsenals with the products of medicinal plants used against inflammation and pain.^42^, ^43^

In the present study, Me‐RCR was first tested for anti‐nociceptive properties using the acetic acid‐induced writhing test and the formalin‐induced pain test. Pain is induced by acetic acid administration and writhing is initiated when free arachidonic acid is released from tissue phospholipids through biosynthesis of prostaglandins (PGs) resulting in a local inflammatory response.^44^ The increased PG levels in the peritoneal cavity boost the pain‐sensation by rising capillary permeability.^7^, ^45^ Substances capable of reducing the level of writhing can exert anti‐nociceptive effects by reducing the synthesis of prostaglandin – a peripheral mechanism of pain inhibition.^44^ The intraperitoneal administration of Me‐RCR significantly (*P* < .05) reduced the writhing index, which might be the result of analgesic compounds acting along with PG analogues.

The formalin‐induced paw licking test is characterized by two distinct pathways – early and late phase pathways. In the early phase, pain, termed neurogenic pain, is induced just after formalin administration. In this phase, pain is associated with the direct stimulation of nociceptive neurons through the release of bradykinin and substance P.^6^, ^29^, ^46^ The late phase, on the other hand, is initiated within 15 minutes of formalin injection, and is associated with the actions of PG, bradykinin, serotonin, and histamine in peripheral tissues.^47^ A significant reduction in pain sensation following ME‐RCR treatment was observed in both early and late phases, but the effect was more pronounced in the late phase. The inhibitory effect of *C. recurvata* on the late phase of the formalin‐licking test indicates the anti‐inflammatory action of the extract. Opioid analgesics are reported to be able to inhibit both phases, with the predominant action in early phase, whereas peripheral analgesics mostly reduce the late phase.^48^ Hence, the reduction of licking time in both the phases with Me‐RCR is an indication of both central and peripheral pain modulation.

There are numerous established mechanisms that can elicit the etiology of diarrhea such as increased secretion of water and electrolytes (secretory diarrhea), infection or inflammation‐induced mucosal injury (exudative diarrhea), and unbalanced intestinal motility (motility diarrhea).^49^ Castor oil‐induced diarrhea is characterized by secretory and motility diarrhea. Ricinolic acid is the principle constituent of castor oil and causes local irritation and subsequent inflammation in the intestinal mucosa. PG is released leading to deranged intestinal motility and hypersecretion of water as well as electrolytes.^32^ The ricinoleic acid forms ricinoleate salts with Na^+^ and K^+^ in the lumen of the intestine. These ricinoleate salts inhibit Na^+^‐K^+^ ATPase, and thus increase the permeability of intestinal epithelium and increase the secretion of water and electrolytes.^6^, ^32^ In this study, the significant anti‐diarrhoeal action exerted by the ME‐RCR extract may be related to inhibition of inflammatory mediators or gastrointestinal hypersecretion.

In the PASS prediction analysis, the Pa values of nyasicoside and curculigine were 0.454 and 0.480, respectively, indicating a high probability of activity of these compounds as anti‐nociceptive agents. The molecular docking analysis allowed precise prediction of the interaction of ligands with receptors, as well as the binding energy, which gave a good idea of the interaction between the compounds and the cyclooxygenases. The grid based docking study revealed the binding modes of the candidate molecules with potential amino acids residing in active pockets of the protein.^50^ Analysis of docking between the active compounds of *C. recurvate* and the active sites of both COX‐1 and COX‐2 was performed in an attempt to find a potential lead compound. Of the three the compounds, curculigine and iso‐curculigine had the lowest docking scores with COX‐1 and COX‐2 receptors: −10.434 and −8.868, respectively. Recently, Uddin et al (2018) illustrated that a negative and low binding energy is an indicator of strong binding affinity to the COX‐1 and COX‐2 enzymes.^29^

Molecular docking studies with the M3 muscarinic acetylcholine receptor (PDB ID: 4U14 and 5AIN) showed that all the compounds interacted with amino acid residues through hydrogen bonds and π‐π stacking interactions, with docking scores ranging between −1.64 and −5.32. From the in silico results, it can be concluded that among the phytoconstituents, isocurculigine and nyasicoside are responsible for the anti‐diarrheal activity of Me‐RCR through interaction with these target proteins.

Natural product researchers should aim to carry out virtual screening for previously isolated potentially active molecules that have not yet been assayed against specified drug targets to check for the possibility of good ADME/T profiles.Y^51^ It is clear that among the compounds tested, curculigine satisfied Lipinski's rule of five, with lower toxicity and better pharmacokinetic profiles, and can therefore be considered a potential drug candidate.

## 5 CONCLUSION

The present study showed that a methanol extract of *C. recurvata* rhizome (Me‐RCR) may contribute to anti‐diarrheal and anti‐nociceptive activities in vivo due to the presence of secondary pharmacologically active metabolites. In addition, PASS program analysis of curculigine, isocurculigine, and nyasicoside indicated the anti‐nociceptive potential of the compounds, molecular docking analysis predicted a higher affinity for binding with COX‐1 and COX‐2, and lastly ADME/T analysis revealed better pharmacokinetic and toxicity profiles. Further analysis of the herb is required, which should include HPLC, NMR, and MS to confirm the exact molecule(s) responsible for the aforesaid activities. In‐depth mechanistic investigations should also be performed using animal models, including a dose‐response study to explain the anti‐nociceptive and anti‐diarrheal activities of selected potential compounds.

## CONFLICT OF INTEREST

None.

## AUTHOR CONTRIBUTIONS

SA, NC, AH and MSHK carried out the study. MSN, ASMAR, SI, SMT, AHMKA, MAH and MSIA contributed in the analysis and interpretation of data, and drafting the manuscript. MAH and ASMAR coordinated the research, revised the manuscript and approved the final version to be submitted for publication. All authors read and approved the final manuscript.

## ETHICAL APPROVAL

The protocol used in this study using mice as the animal model for anti‐nociceptive and anti‐diarrheal study was approved by the institutional animal ethics committee, Department of Pharmacy, International Islamic University Chittagong, Bangladesh (ref. IIUC/Pharm‐78/09‐15).
